# Study of High-Performance GaN-Based Trench CAVET with Stepped Doping Microstructure

**DOI:** 10.3390/mi13081273

**Published:** 2022-08-07

**Authors:** Yuan Li, Liang Xu, Zhiyou Guo, Huiqing Sun

**Affiliations:** 1 Institute of Semiconductor Science and Technology, South China Normal University, 55 Zhongshan Avenue, Tianhe District, Guangzhou 510631, China; 2 Foshan NationStar Semiconductor Co., Ltd., Foshan 528226, China

**Keywords:** GaN HEMT, trench current-aperture vertical electron transistor (CAVET), stepped doping, breakdown voltage, specific on-resistance

## Abstract

In this article, an innovative GaN-based trench current-aperture vertical electron transistor (CAVET) with a stepped doping microstructure is proposed and studied using Silvaco-ATLAS. According to the simulation and analyzed characteristics, the best performance renders a remarkable Baliga’s figure of merit (FOM) of 4.767 GW·cm^2^ owing to the modulation of the electric-field distribution. By adjusting the size of the stepped doping microstructure and doping concentration in the GaN drift, the maximum optimized result can achieve a relatively high breakdown voltage (BV) of 2523 V with a very low specific on-resistance (R_on,sp_) of 1.34 mΩ·cm^2^, or the BV can be improved to 3024 V with a specific on-resistance (R_on,sp_) of 2.08 mΩ·cm^2^. Compared with the conventional superjunction GaN-based trench CAVET, the newly demonstrated structure can achieve a 43% reduction in R_on,sp_ and increase by almost 20% the original BV. These results indicate the superiority of using the stepped doping microstructure in a trench CAVET to improve the BV and decrease R_on,sp_, providing a reference for further development of GaN-based CAVETs.

## 1. Introduction

Over the past few decades, GaN-based devices went through rapid development, such as GaN MOSFETs, GaN Schottky diodes [1] and GaN photodetectors [2]. In the field of high-power applications, GaN-based power devices with a vertical structure attract much attention for their suitability in the fabrication of high-efficiency power converters [3]. Although typical lateral structures [4,5] dominate the GaN-based high-electron-mobility transistors (HEMTs), vertical devices holding a vertical conduction path draw great interests for their superior ability in achieving high breakdown voltages (BVs) and high current levels without enlarging the chip size or introducing extra thermal management [6,7], such as GaN vertical MOSFETs [8], GaN interlayer-based vertical MOSFETs [9] and GaN-based trench current-aperture vertical electron transistors (CAVETs) [10]. Among these, the trench CAVET deserves attention since it enables the normally off mode to be easily realized, compared with planar-gate CAVETs [11]. In addition, the high-electron-mobility channels formed by polarization-based 2DEG in GaN-based CAVETs make them more suitable for applications in the order of kilowatts [7]. In 2013, Li [10] et al. proposed an enhancement-mode CAVET with a superjunction (SJ). The design of a half p-GaN pillar below the current blocking layer (CBL) greatly improved the BV of the device. The best R_on,sp_ of 4.2 mΩ·cm^2^ with a BV of 12.4 kV was obtained. In 2016, Shibata [11] et al. reported a CAVET with a regrown p-GaN/AlGaN/GaN trench-gate semi-polar structure. Making possible normally off operation, the CAVET held a specific on-resistance (R_on,sp_) of 1.0 mΩ·cm^2^ and a BV of 1.7 kV. In 2018, Ji [12] et al. successfully fabricated an MIS trench-gate CAVET with a high BV of 880 V and low R_on,sp_ of 2.7 mΩ·cm^2^. By adopting standard GaN-HEMT epitaxial high temperature, the high-quality in situ Si_3_N_4_ gate dielectric brought a low hysteresis of ~0.1 V. In 2022, Bai [13] et al. simulated a novel trench-gate CAVET with dual-current aperture. By inserting the p-GaN island between the two p-GaN CBLs, the electric-field distribution was properly modulated to achieve a high BV of 1504 V and low R_on,sp_ of 0.77 mΩ·cm^2^.

In conventional planar MOSFETs, R_on,sp_ has a weak correlation with the BV [14], whereas vertical structures such as SJ-HEMTs and CAVETs establish a linear relationship between R_on,sp_ and the BV [15]. CAVETs are specialized in high-voltage and high-power applications, especially when introducing an SJ, which is a bulk of p-buried layers to deplete the 2DEG in the channel. The geometry, thickness and doping are significant parameters to determine the forward characteristics and balance the tradeoff between R_on,sp_ and the BV. However, while the BV can be improved utilizing the P-buried layer since it can fully deplete the 2DEG in the channel [16], it also increases R_on,sp_ and reduces the maximum saturation current due to the decrease in the 2DEG density of the channel, which degrade the forward characteristics of the device. As per the principle of charge compensation, modulating the drift region in CAVETs using a uniform doping profile is a feasible method to surpass the performance of conventional SJ-CAVETs. Yet, seldom investigations and experiments were carried out on GaN-based CAVETs using p-buried layers or SJs in the drift. Although a few simulations [17,18] designed nonuniform doping profiles in the drift of GaN-based CAVETs, these studies only focused on the half pillar of the SJ and its doping variation in the longitudinal direction. There are no research studies reporting about the alternation in the drift doping region along the plane axis. Thus, the purpose of this paper is to design a reliable GaN-based CAVET model to explore and predict the influence of nonuniform profile doping in the horizontal direction. 

In this paper, a GaN-based trench CAVET with stepped doping microstructure is proposed to further improve both the breakdown voltage (BV) and specific on-resistance (R_on,sp_). By alternating the size and doping concentration of n-, p-buried regions in the GaN drift layer, the 2DEG can be appropriately depleted in the channel, and the vertical excess electric field from source to drain can be absorbed as an extra current blocking layer (CBL). Meanwhile, the current density in the channel can be appropriately promoted, and the electric field in the vertical path can be boosted. Utilizing this method, the increase in specific on-resistance can be ignored, while the BV can be remarkably enhanced. Thus, the option of modulating the GaN drift layer via stepped doping is a feasible choice to achieve a trade-off performance enhancement. To evaluate the performance and investigate the parameters, two-dimensional numerical simulations are operated and analyzed using Silvaco-Atlas.

## 2. TCAD Model Simulation

**Figure 1:**
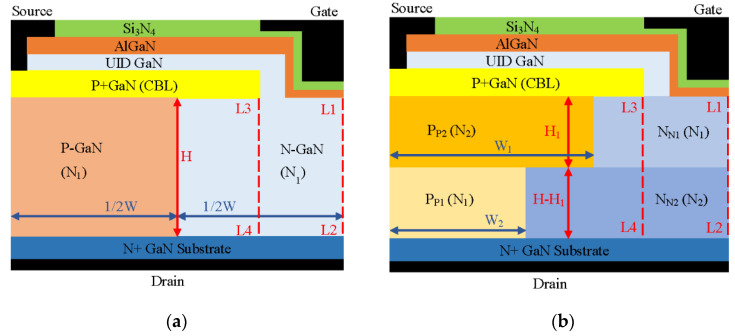
Schematic diagrams of (**a**) SJ-Trench CAVET and (**b**) SDS-Trench CAVET. Lines L1L2 and L3L4 indicate the vertical path along device center and CBL, respectively.

The half-cell schematic cross-section of a GaN-based trench CAVET with stepped doping microstructure (SDS-Trench CAVET) is illustrated in Figure 1b. In addition, a superjunction GaN-based trench CAVET (SJ-Trench CAVET) is also exhibited in Figure 1a for comparison. With an overall width of 5 μm, the main parameters include a 2 μm thick GaN n+ substrate, a 15 μm thick n-GaN drift layer and a 700 nm thick p+ GaN current blocking layer (CBL). The channel layer is composed of 20 nm thick Al_0.25_Ga_0.75_N and 210 nm thick UID GaN. In addition, 60 nm thick silicon nitride (Si_3_N_4_) serves as a gate dielectric. The gate length, trench depth and CBL length are set as 2 μm, 1 μm and 4 μm, respectively. The n+ GaN substrate is heavily doped with Si, and the p+ GaN is heavily doped with Mg. On the basis of Ref. [19], the origin CAVET is redesigned, and the other detailed device parameters are represented in Table 1.

As is shown in Figure 1a, half of the GaN drift layer in the SJ-Trench CAVET is divided into an n-pillar and a p-pillar with the same width (1/2W) and the same height (H). In the SDS-Trench CAVET, the GaN drift layer is evenly separated into a top stepped doping microstructure and a bottom stepped doping microstructure. In addition, both the top part and bottom part of the stepped doping microstructure are divided into two parts, the p-doping area below the CBL and the n-doping area adjacent to it. The same doping concentration (N_1_) is used in both top n-doping part N_N1_ and bottom p-doping part P_P1_. Simultaneously, the same doping concentration (N_2_) is used in both bottom n-doping part N_N2_ and top p-doping part P_P2_. Doping concentration increment N_s_ is defined as N_2_ minus N_1_. In addition, doping concentration N_1_ in the SDS-Trench CAVET is initially set equal to that of the n- and p-pillars in the SJ-Trench CAVET. For simplicity, typical N_1_ concentrations, namely, from 0.5 × 10^16^ cm^−3^ to 2 × 10^16^ cm^−3^, are utilized in simulations. Top part N_N1_ and bottom part P_P1_ keep the same width (W_2_); thus, bottom part N_N2_ and top part P_P2_ are given the same width (W_1_). It is worth noting that W_1_ should not exceed the length of the CBL to avoid excessive depletion of 2DEG in the channel.

To obtain more accurate results in Silvaco TCAD, the concentration dependent (CONSRH) and Auger physical models are used as recombination models, while Selberherr’s Model (IMPACT SELB) is adopted for impact ionization. In addition, the high-field-saturation and -polarization models are also used in the simulation. According to Ref. [10], Li et al. proposed and simulated a classic GaN vertical superjunction HEMT. Thus, the model and material parameters could refer to a similar device. The parameter details of low-field-mobility models and Farahmand-Modified Caughey–Thomas (FMCT) models are listed in Table 2.

When the breakdown performance is simulated, the avalanche model with a dependent electric field is adopted in the simulations. The impact-ionization rate is defined by [20]:

(1) $$G=\alpha_{n}n\nu_{n}+\alpha_{p}p\nu_{p}$$

In Equation (1), n and p refer to the concentrations of electrons and holes, respectively, while $\nu_{n}$ and $\nu_{p}$ refer to the saturation velocities of electrons and holes, respectively, where $\alpha_{n}$ and $\alpha_{p}$ are the values of the impact-ionization coefficient related to the electric field. Equations (2) and (3) define $\alpha_{n}$ and $\alpha_{p}$ as follows:

(2) $$\alpha_{n}=\gamma_{n}a_{n}\exp\left(-b_{n}\gamma_{n}/E\right)$$

(3) $$\alpha_{p}=\gamma_{p}a_{p}\exp\left(-b_{p}\gamma_{p}/E\right)$$

In Equations (2) and (3) the temperature dependence of the phonon gas against which carriers are accelerated is expressed by parameters $\gamma_{n}$ and $\gamma_{p}$. Coefficients $a_{n}$, $a_{p}$, $b_{n}$ and $b_{p}$ are fitting parameters, with modeling parameters $a_{n}$ = 2.81 × 10^8^ cm^−1^, $b_{n}$ = 3.43 × 10^7^ V/cm, $a_{p}$ = 5.41 × 10^6^ cm^−1^ and $b_{p}$ = 1.96 × 10^7^ V/cm [21].

**Figure 2:**
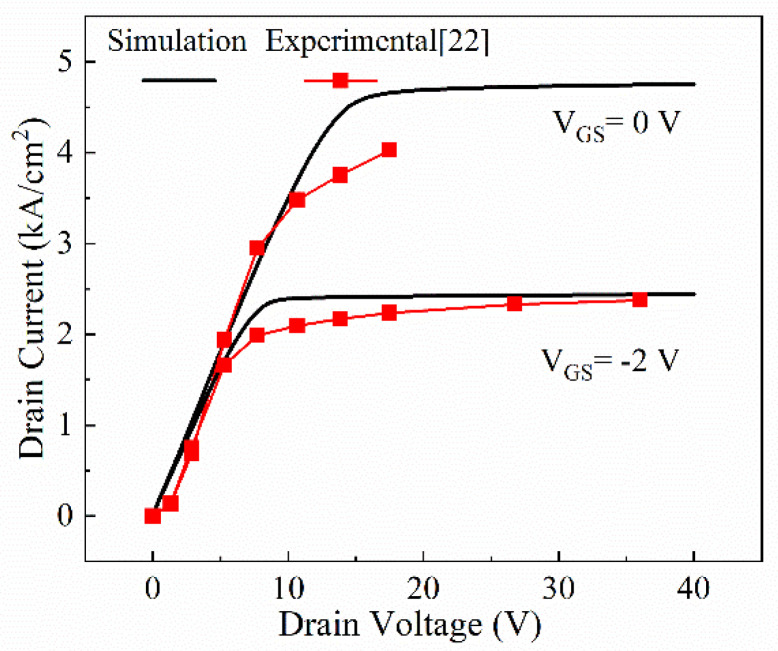
Experimental- [22] and numerical-result comparison of on-state I–V characteristics.

In order to examine the validity of the proposed model and parameters, Figure 2 compares the experimental results from Chowdhury [22] et al. and the simulation results when the normally on CAVET is in the on state at V_GS_ = 0, −2 V. The reference is similar to the designed CAVET; thus, these models are considered available in simulations. As shown in Figure 2, the I_DS_–V_DS_ curve shows a great fit between the experimental data and the simulation results. In addition, R_on,sp_ also obviously indicates a good agreement between them. Thus, the models adopted are proved to be effective and correct.

## 3. Results and Discussion

**Figure 3:**
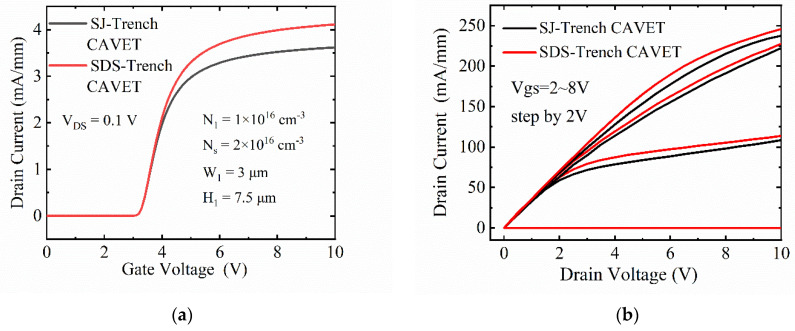
Comparison of on-state I–V characteristics for two structures, including (**a**) transfer curves and (**b**) output curves.

Figure 3 juxtaposes the transfer characteristics of the two devices with their output characteristics. In Figure 3a, the same threshold voltage (V_th_) of 3.1 V is manifested in the SDS-Trench CAVET and SJ-Trench CAVET. However, compared with the SJ-Trench CAVET, the drain current of the SDS-Trench CAVET prevails when the gate voltage goes beyond V_th_, which indicates a smaller resistance in the proposed structure. Furthermore, in Figure 3b, the noticeable enhancement of the on-state I–V curve demonstrates that the R_on,sp_ value of 2.08 mΩ·cm^2^ in the SDS-Trench CAVET is smaller than that of 2.37 mΩ·cm^2^ in the SJ-Trench CAVET. 

**Figure 4:**
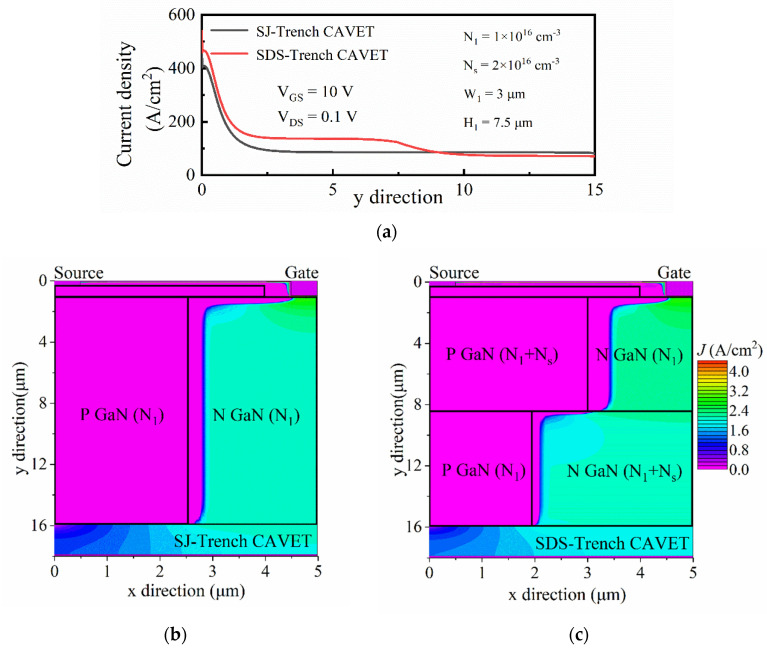
(**a**) On-state current-density curve of two devices along line L1L2 and (**b**,**c**) corresponding total two-dimensional distribution for SJ-Trench CAVET and SDS-Trench CAVET under the conditions of V_GS_ = 10 V and V_DS_ = 0.1 V (in log scale).

To explain the upgraded performance of the SDS-Trench CAVET, the on-state current-density distributions are analyzed in Figure 4. For both devices, only the n-doping area in the drift is conductive, since n+ substrate/p-GaN is under reversed bias as well as lateral n/p connection [10,23]. As shown in Figure 3b,c, the horizontal current paths are exhibited in the SDS-Trench CAVET and SJ-Trench CAVET. Obviously, in the SDS-Trench CAVET, N_N1_ owns a narrower current path, but N_N2_ holds a wider current path, owing to the varied width of the stepped doping part. However, the current density in N_N1_ is greater than that in the corresponding position in the SJ-Trench CAVET, which is attributed to the extended P_P1_ with a higher doping concentration, making the electric charge accumulate in N_N1_. From Figure 4a, a greater current density along line L1L2 can be seen in the n-doping area of the SDS-Trench CAVET. It can be noticed that the current-density curve in N_N2_ is slightly lower than that in the corresponding position in the SJ-Trench CAVET. As for the reason, the higher doping concentration in N_N2_ may bring higher current density, but the electrons disperse in the wider N GaN drift. Consequently, with a spacious current direction and a high current density, the proposed device represents the potential to modulate current density and promote current conduction.

**Figure 5:**
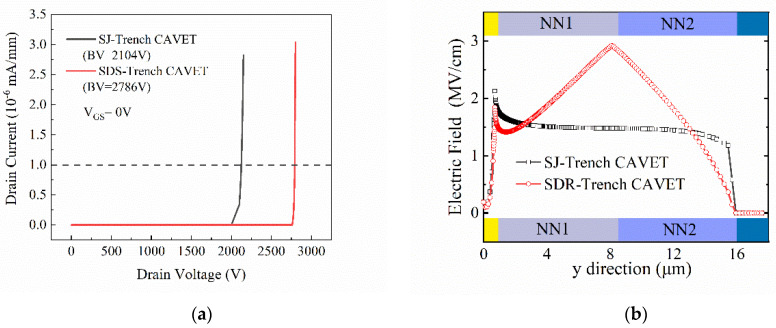
(**a**) Off-state BV curves of SJ-Trench CAVET and SDS-Trench CAVET and (**b**) electric-field strength comparison in the GaN drift along line L3L4.

**Figure 6:**
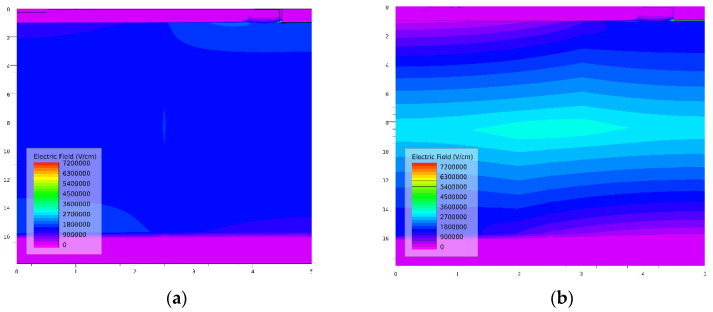
Corresponding entire-electric-field contour plots of (**a**) SJ-CAVET and (**b**) SDS-CAVET

From the corresponding off-state breakdown characteristics in Figure 5a, the BVs of the SJ-Trench CAVET and SDS-Trench CAVET extracted at I_D_ = 1 × 10^−6^ mA/mm are 2104 V and 2786 V, respectively. To investigate the distinction between the two devices and mechanisms accounting for it, the electric field and the corresponding contour plots are illustrated in Figure 5b and Figure 6, respectively. Figure 5b represents the comparison of the electric-field strengths along line L3L4 in the proposed structures. As can be seen, owing to the incomplete depleted 2DEG in the trench, there are two small peaks of electric field in the corresponding position. However, the larger p-GaN region below the CBL in the SDS-Trench CAVET consumes more electric field in the trench corner, resulting in a lower peak at the CBL/GaN drift interface. Additionally, since N_N2_ is given a higher doping concentration and a larger region, the electric-field strength is enhanced and reaches its maximum at the dividing line. In addition, the electric-field strength approaching the substrate is smaller in the SDS-Trench CAVET, which could be explained by the fact that the electric field is distributed and weakened in the larger N_N2_. The greater value of the electric field in the SDS-Trench CAVET results in the tolerance to higher forward voltage [24]. Figure 6 plots more details about the two peaks and the whole distribution in the GaN drift. In the SJ-CAVET, there are only two strong electric fields, which are located in the trench corner and substrate. However, the drift layer in the SDS-CAVET is much brighter than the former, especially at the stepped doping interface. Such a great electric-field strength guarantees the high current levels and high voltage tolerance of the device.

**Figure 7:**
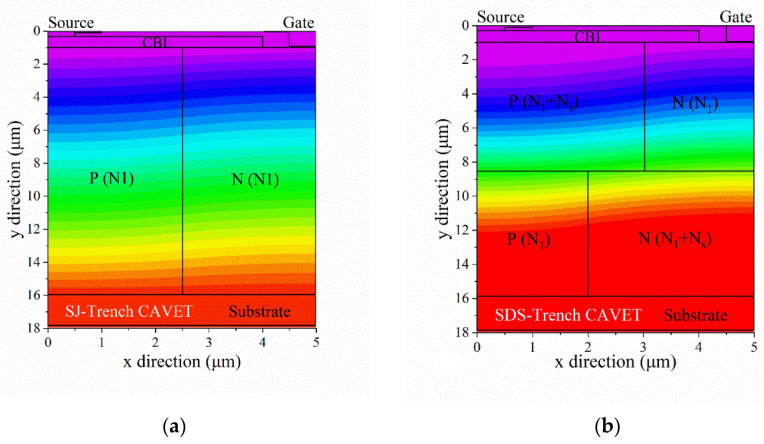
Off-state performance of (**a**) SJ-Trench CAVET compared with (**b**) SDS-Trench CAVET according to potential equipotential surface.

In Figure 7, the off-state potential equipotential lines show similar slopes in the SJ-Trench CAVET and SDS-Trench CAVET, which guarantee device stability in breakdown performance. Compared with the SJ-Trench CAVET, in the SDS-Trench CAVET, the low-potential areas below the CBL move down slightly, and the high-potential areas above the substrate move upward a lot, causing narrower medium-potential areas. The variation in equipotential-line densities verifies the electric-field-distribution result in Figure 5, whereby the electric-field strength near the middle is enhanced, while that near the bottom is degraded in n- and p-pillars. The total enlarged high-potential distribution in the bottom area is beneficial for breakdown performance, especially during reverse blocking.

**Figure 8:**
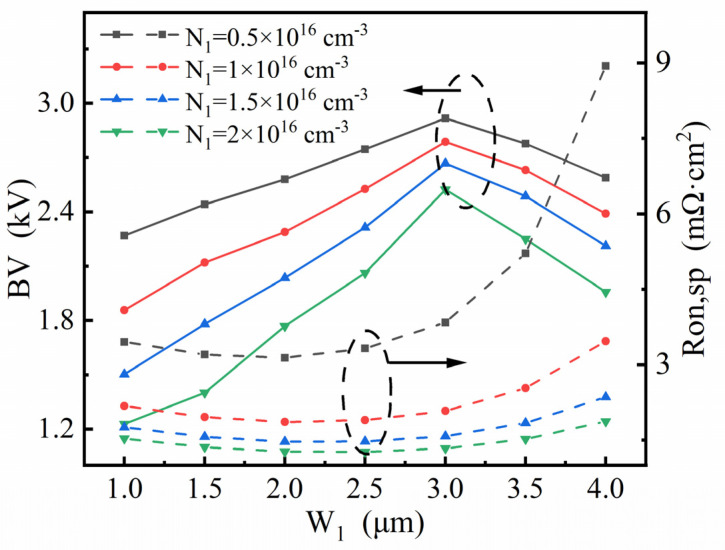
Investigation of W_1_ affecting optimized BV and R_on,sp_ in SDS-Trench CAVET for varied values of N_1_ for a given N_s_ = 2 × 10^16^ cm^−3^.

**Figure 9:**
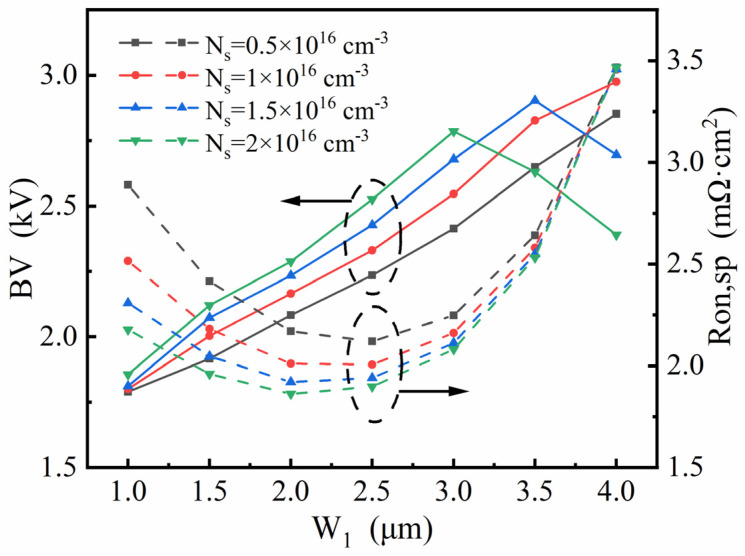
Investigation of W_1_ affecting optimized BV and R_on,sp_ in SDS-Trench CAVET for varied values of N_s_ for a given N_1_ = 1 × 10^16^ cm^−3^.

**Figure 10:**
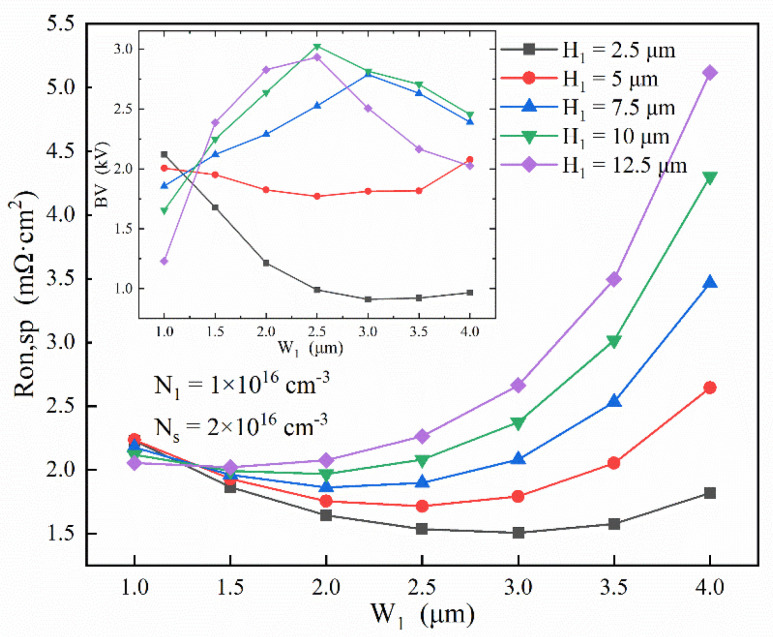
Investigation of H_1_ and W_1_ affecting optimized BV and R_on,sp_ in SDS-Trench CAVET for varied values of N_1_ for given N_s_ = 2 × 10^16^ cm^−3^ and N_1_ = 1 × 10^16^ cm^−3^.

Figure 8, Figure 9 and Figure 10 show how the four key parameters, N_1_, N_s_, H_1_ and W_1_, influence the breakdown characteristics and R_on,sp_. Since the stepped doping region is composed of top part and bottom parts, step lengths W_1_ and W_2_ deserve investigation to explore their modulation effect and are consequently selected as the main variations in the optimization. Figure 8 shows the plots of the breakdown voltage and specific on-resistance versus length W_1_ for different values of N_1_ for given N_s_ and H_1_ in the SDS-Trench CAVET. As shown, R_on,sp_ is at its minimum when W_1_ is 2.0 μm or 2.5 μm, while the maximum BV appears when W_1_ is 3 μm. Although the largest BV is obtained with N_1_ = 0.5×10^16^ cm^−3^, its R_on,sp_ exceeds the original R_on,sp_ in the SJ-Trench CAVET. Thus, the best BV result, 2786 V, is obtained with N_1_ = 1 × 10^16^ cm^−3^ with a slightly reduced R_on,sp_ of 2.08 mΩ·cm^2^, or a lower R_on,sp_ of 1.34 mΩ·cm^2^ and a relatively high BV of 2500 V could be acquired when N_1_ = 2 × 10^16^ cm^−3^. The above two trade-offs are both represented with W_1_ = 3 μm, which means that such a stepped doping method is effective and significant in enhancing the performance of the devices.

In Figure 9, for given N_1_ and H_1_, the different values of N_s_ are investigated to further examine the above results. The peak of the BV emerges when W_1_ is 3 μm or longer. While the BV increases as N_s_ declines, the rapidly enlarged R_on,sp_ cannot be ignored. Therefore, the optimized result is achieved with N_s_ = 2 × 10^16^ cm^−3^, since it represents a higher BV and lower R_on,sp_ than other N_s_, especially when W_1_ is smaller than 3 μm.

Based on the above results, Figure 10 explores how the thickness of the stepped doping structure affects the performance of the SDS-Trench CAVET with given N_s_ = 2 × 10^16^ cm^−3^ and N_1_ = 1 × 10^16^ cm^−3^. It seems that R_on,sp_ follows a regular pattern, whereby as W_1_ is expanded, R_on,sp_ first decreases and then increases. In addition, when H_1_ is enlarged, R_on,sp_ rapidly declines, which could be ascribed to the overconsumption of 2DEG by the broadened p-doping region. However, in the embedded figure of BV variation, the trend is not the same as that of R_on,sp_. As is shown, when W_1_ is extended, the BV first increases and then drops when H_1_ is larger than 5 μm, while the BV seldom changes or even descends when H1 is smaller than 5 μm. Pursuing the highest BV, 10 μm H_1_ provides a 3024 V breakdown voltage with a non-deteriorated R_on,sp_ of 2.08 mΩ·cm^2^, which is obtained when W_1_ = 2.5 μm. Such a high breakdown voltage could be attributed to the thickened p-doping region beneath the CBL, greatly affecting the reverse-blocking process [25].

## 4. Fabrication Process

Recent years witnessed more and more mature GaN-growth technologies, such as technologies for thick in situ doping-GaN-layer growth, GaN-based selective-area-growth technologies (SAG) and GaN etching technologies. The height of the GaN drift layer grown via SAG and the depth of the plasma-etched GaN trench were reported to be more than 10 μm [26]. Therefore, the fabrication of the proposed GaN-based trench CAVET with a stepped doping microstructure is achievable, since the n-doping region and p-doping region could alternately be grown using the above technologies.

**Figure 11:**
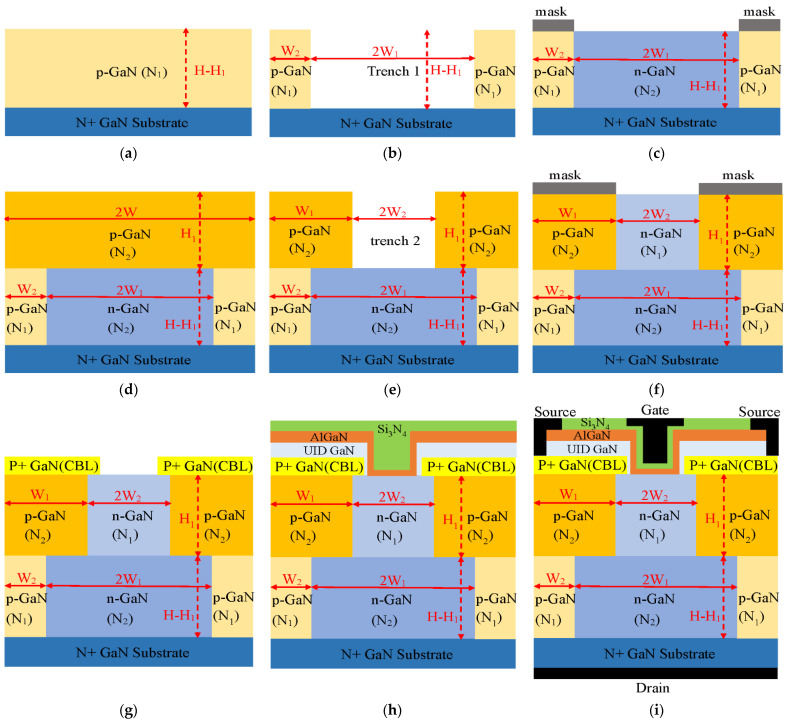
A feasible process to realize the proposed SDS-Trench CAVET, showing (**a**) bottom-p-GaN-layer growth, (**b**) trench 1 etching, (**c**) bottom-n-GaN-layer regrowth in trench 1, (**d**) top-p-GaN-layer growth, (**e**) trench 2 etching, (**f**) top-n-GaN-layer regrowth in trench 2, (**g**) CBL-layer growth and etching, (**h**) UID-GaN/AlGaN/Si_3_N_4_ triple-trench-layer regrowth and (**i**) selective etching to form electrode and complete fabrication.

The diagrams in Figure 11 and the following procedures demonstrate an available process to realize the proposed SDS-Trench CAVET. Firstly, a p-GaN layer with doping concentration N1 and height H minus H_1_ is epitaxially grown via metal–organic chemical vapor deposition (MOCVD) on a bulk conductive GaN substrate. Mg ions could be implanted into the bottom p-GaN layer at 80 keV [22]. Utilizing the Cl-based inductively coupled plasma (ICP) etching process, trench 1 with width 2W_1_ and height H–H_1_ is formed. Then, the rest of the p-GaN layer is masked and an n-GaN layer with doping concentration N_2_ and height H–H_1_ is regrown via SAG in trench 1. Secondly, a p-GaN layer with doping concentration N_2_ and height H_1_ is epitaxially deposited via MOCVD. The same Mg ion-implantation method is applied to it. Simultaneously, trench 2 is etched via ICP, and the n-GaN layer with doping concentration N_1_ and height H_1_ is regrown via SAG to fill trench 2. Thirdly, a heavily Mg-implanted doped p-GaN layer is epitaxially grown via MOCVD to form a CBL layer. The current aperture is protected using an implantation mask consisting of Ti/Ni on SiO_2_ deposited via PECVD. The implantation mask is later removed with the wet-etching technique using hydrofluoric acid. After wet etching and MOCVD growth on the CBL layer, the final main procedures involve the deposition of the UID-GaN channel and MIS trench-gate structure. After p+GaN activation and HF surface treatment, unintentionally doped (UID) GaN is regrown on the CBL via MOCVD. Using Cl_2_/BCl_3_ gases in reactive-ion etching (RIE) to etch the trench, UV–ozone and HF cleaning treatments are performed after trench formation [12]. Subsequently, the AlGaN layers are grown via MOCVD at a high temperature. The Si_3_N_4_ dielectric is deposited on the AlGaN barrier layer via PECVD. Then, the trench gate and source are formed via ICP etching, followed by surface treatment. Next, the buried p-GaN and CBL are processed via thermal annealing with the diffusion of hydrogen and Mg activation. Later, the dielectric gate is patterned via ALD. Finally, the Ni/Au stack is taken as the gate, while the Ti/Al stack is used to form the source and backside drains, after which the entire proposed device is obtained.

## 5. Conclusions

A GaN-based trench CAVET with a stepped doping microstructure is demonstrated and evaluated using two-dimensional simulations to promote the BV and R_on,sp_. By modulating the step width, thickness, doping concentration and variation in the GaN drift layer, the structure presents an extremely high BV of 3024 V with R_on,sp_ of 2.08 mΩ·cm^2^. Defining Baliga’s figure of merit (FOM) as BV^2^/R_on,sp_, the optimized device achieves a high FOM of 4.396 GW·cm^−2^, or the device can manifest an extremely low R_on,sp_ of 1.34 mΩ·cm^2^ with a BV of 2523 V, yielding a remarkable FOM of 4.767 GW·cm^−2^. The above two trade-offs exhibit the potential and superiority of the stepped doping microstructure in enhancing the performance of high-power GaN devices and applications.

## Figures and Tables

**Table 1:** Key device parameters set in simulation [19].

Symbol	Statement	Value
L_CBL_	CBL length	4 um
N_CBL_	CBL doping concentration	1 × 10^18^ cm^−3^
H	Total GaN-drift-layer thickness	15 μm
H_1_	Thickness of P_P2_ and N_N1_	2.5 μm~12.5 μm
W	Total GaN-drift-layer length	5 μm
W_1_	Width of P_P2_ and N_N2_	1 μm~4 μm
W_2_	Width of P_P1_ and N_N1_	W minus W_1_
T_S_	n+ GaN substrate thickness	2 μm
N_S_	n+ GaN substrate doping concentration	1 × 10^19^ cm^−3^
D_T_	Trench depth	1 μm
L_G_	Gate length	2 μm
N_1_	Doping concentration in N_N1_ and P_P1_	0.5 × 10^16^~2 × 10^16^ cm^−3^
N_2_	Doping concentration in N_N2_ and P_P2_	N_1_ + N_S_

**Table 2:** Material parameters used in simulation.

Statement	Parameter	Material	Value
Electron mobility	MUN	AlGaN	300 cm^2^/(V·s)
Hole mobility	MPN	AlGaN	10 cm^2^/(V·s)
Lifetime of electron	TAUN0	AlGaN	1 × 10^−8^ s
Lifetime of hole	TAUP0	AlGaN	1 × 10^−8^ s
FMCT electron-mobility model	MU1N.FMCT	GaN	55 cm^2^/(V·s)
FMCT electron-mobility model	MU2N.FMCT	GaN	1000 cm^2^/(V·s)
FMCT electron-mobility model	NCRITN.FMCT	GaN	2 × 10^17^
FMCT electron-mobility model	ALPHAN.FMCT	GaN	1.0
FMCT hole-mobility model	MU1P.FMCT	GaN	3 cm^2^/(V·s)
FMCT hole-mobility model	MU2P.FMCT	GaN	170 cm^2^/(V·s)
FMCT hole-mobility model	NCRITP.FMCT	GaN	3 × 10^17^
FMCT hole-mobility model	ALPHAP.FMCT	GaN	2.0
